# Performance of common fetal growth standards in pregnancies affected by systemic lupus erythematosus

**DOI:** 10.1590/1806-9282.20260076

**Published:** 2026-06-22

**Authors:** Ana Carolina Rei Pereira Barros, Gustavo Yano Callado, Edward Araujo, Rodrigo Rocha, Nilson Ramires de Jésus, Guilherme Ramires de Jésus, Fernando Maia Peixoto-Filho

**Affiliations:** 1 Universidade do Estado do Rio de Janeiro, Department of Gynecology and Obstetrics – Rio de Janeiro (RJ), Brazil.; 2 Faculdade Israelita de Ciências da Saúde Albert Einstein, Discipline of Woman Health – São Paulo (SP), Brazil.; 3 Universidade Federal de São Paulo, Escola Paulista de Medicina, Department of Obstetrics – São Paulo (SP), Brazil.; 4 Universidade Municipal de São Caetano do Sul, Discipline of Woman Health – São Caetano do Sul (SP), Brazil.

**Keywords:** Systemic lupus erythematosus, Fetal growth restriction, Fetal weight, Ultrasonography, Pregnancy outcomes

## Abstract

**OBJECTIVE:**

The aim of this study was to compare the performance of different fetal growth reference standards—Hadlock, INTERGROWTH-21st, and World Health Organization—in pregnancies complicated by systemic lupus erythematosus.

**METHODS:**

This retrospective cohort study was conducted at a tertiary referral center and included singleton pregnancies delivered at ≥22 weeks of gestation. Pregnant women were classified into two groups: those with systemic lupus erythematosus, defined by ≥4 American College of Rheumatology criteria, and controls without autoimmune disease. Estimated fetal weight obtained from routine obstetric ultrasonography was converted into gestational age-specific percentiles using the Hadlock, INTERGROWTH-21st, and World Health Organization reference standards. Correlations between ultrasound-based fetal weight percentiles and birth weight percentiles were assessed using Spearman's rank correlation coefficient.

**RESULTS:**

Of 225 initially identified pregnancies, 179 fetuses were included in the final analysis, comprising 124 from women with systemic lupus erythematosus (69.3%) and 55 from controls (30.7%). Data distribution was non-normal. Across all growth standards, fetuses from women with systemic lupus erythematosus demonstrated lower mean weight percentiles than those from controls. Mean percentiles for controls versus the systemic lupus erythematosus group were 47.1% versus 38.4% using Hadlock, 63.3% versus 55.2% using INTERGROWTH-21st, and 51.7% versus 42.6% using World Health Organization. No statistically significant differences were observed among the three reference standards; however, INTERGROWTH-21st consistently yielded higher percentile values in both groups.

**CONCLUSION:**

Although fetal weight classification varied according to the growth standard used, pregnancies complicated by systemic lupus erythematosus consistently exhibited lower fetal weight percentiles. Awareness of systematic differences among commonly used growth references is essential, as these may influence the detection of fetal growth restriction.

## INTRODUCTION

Maternal systemic lupus erythematosus (SLE) is associated with a broad spectrum of adverse maternal, obstetric, and fetal outcomes. According to the Society for Maternal-Fetal Medicine, pregnancies complicated by SLE carry a substantially increased risk of preeclampsia (15–35%), fetal growth restriction (FGR) (6–35%), preterm birth (19–49%), pregnancy loss—including miscarriage and stillbirth—and intrauterine fetal death. These adverse outcomes are largely attributed to placental insufficiency, characterized by reduced placental size and vascular lesions such as decidual vasculopathy, thrombosis, and placental infarctions^1,2^.

Accurate assessment of fetal growth through prenatal ultrasonography is a cornerstone of antenatal surveillance, aiming to identify fetuses at increased risk of perinatal morbidity and mortality^3^. Early recognition of impaired fetal growth allows for closer monitoring, timely intervention, and optimization of perinatal outcomes^4^. However, despite the clinical relevance of fetal growth assessment in high-risk pregnancies, there remains no consensus regarding the most appropriate fetal weight reference standard for use in pregnancies complicated by SLE.

Different fetal growth standards have been developed based on distinct populations and methodological approaches, which may lead to variability in fetal weight classification and in the identification of growth abnormalities^3,4^. In pregnancies affected by chronic inflammatory and vascular conditions such as SLE, the choice of growth reference may have important implications for the detection of FGR and subsequent clinical management.

The primary objective of this study was to evaluate the agreement between ultrasound-based estimated fetal weight (EFW) percentiles and birth weight percentiles using different fetal growth standards in pregnancies complicated by SLE and in a control population without autoimmune disease.

## METHODS

### Study design and population

This was a retrospective cohort study conducted at the Perinatal Unit of the Pedro Ernesto University Hospital, State University of Rio de Janeiro (UERJ), Brazil. Medical records from January 2016 to December 2025 were reviewed. This study was approved by the Ethics Committee of UERJ (CAAE: 50726115.4.0000.5259). Due to the retrospective design based exclusively on review of medical records and the absence of direct patient contact, the requirement for written informed consent was waived by the ethics committee.

The study population comprised pregnant women divided into two groups: (1) women with a diagnosis of SLE and (2) women without autoimmune diseases, who served as the control group. Only singleton pregnancies with delivery at or beyond 22 weeks of gestation were eligible for inclusion.

Women in the SLE group were required to meet at least four American College of Rheumatology classification criteria for SLE^5^. The control group included pregnant women with no diagnosis of autoimmune disease.

To minimize potential confounding factors affecting fetal growth, pregnancies complicated by maternal conditions known to be associated with FGR, such as hypertensive disorders of pregnancy, chronic hypertension, diabetes mellitus, renal disease, or other maternal vasculopathies, were excluded.

The control group consisted of low-risk pregnant women without autoimmune disease and without relevant maternal comorbidities.

**Figure 1:**
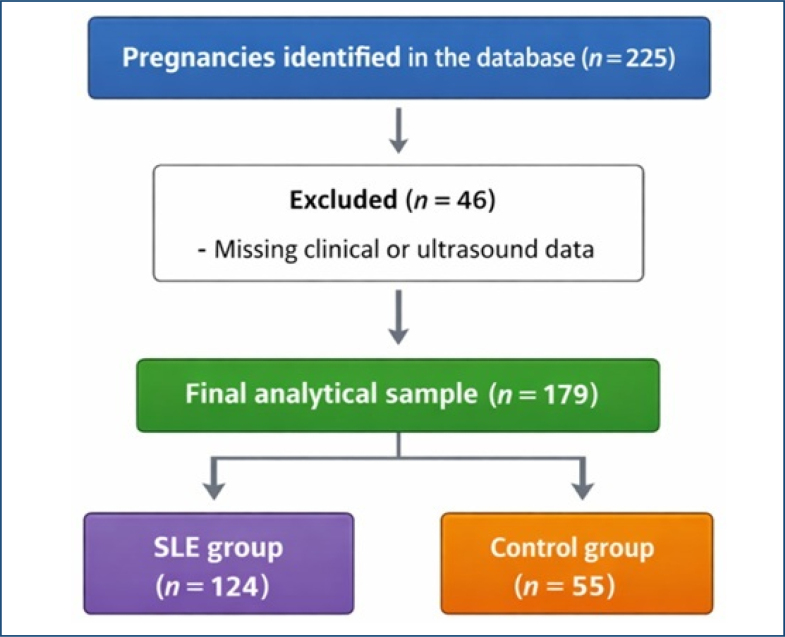
Patient selection.

A total of 225 fetuses met the inclusion criteria, including 157 fetuses from women with SLE and 68 fetuses from women without autoimmune disease (Figure 1). This study was based exclusively on a retrospective review of medical records. Patient confidentiality was strictly maintained throughout data collection and analysis. The protocol was conducted in accordance with institutional ethical standards and applicable national regulations.

This study was conducted and reported in accordance with the Strengthening the Reporting of Observational Studies in Epidemiology guidelines.

### Data collection

Data were obtained through systematic medical record review using a semi-structured data collection form. Maternal demographic characteristics, pregnancy information, ultrasound findings, and neonatal outcomes were extracted from institutional electronic and paper records. Gestational age was established based on first-trimester ultrasonography or, when unavailable, on the date of the last menstrual period corroborated by early second-trimester ultrasound.

### Fetal biometry and estimated fetal weight

Serial obstetric ultrasound examinations were performed as part of routine prenatal care. Fetal biometry included biparietal diameter, head circumference, abdominal circumference, and femur length. EFW was calculated using standard ultrasound-based formulas. The EFW obtained at ultrasound was converted into gestational age-specific percentiles using three different fetal growth reference standards: Hadlock^6^, Intergrowth-21st^7^, and World Health Organization (WHO)^8^. Each fetus was classified independently according to each reference curve.

Small for gestational age (SGA) was defined as birth weight below the 10th percentile for gestational age according to each reference standard. FGR was defined according to the same percentile threshold (<10th percentile) for EFW or birth weight based on the evaluated growth curves.

For the purposes of this analysis, the last ultrasound examination performed prior to delivery was used to obtain the EFW percentile. The interval between this ultrasound examination and delivery was recorded in order to allow comparison between EFW and birth weight percentiles.

### Statistical analysis

Continuous variables were summarized using appropriate measures of central tendency and dispersion, according to their distribution. Categorical variables were expressed as absolute frequencies and percentages. Correlation between EFW percentiles at ultrasound and birth weight percentiles was assessed using Spearman's rank correlation coefficient, given the non-parametric distribution of the data. All statistical analyses were performed using the Statistical Package for the Social Sciences, version 29.0 (Chicago, IL, USA). A two-sided p<0.05 was considered statistically significant.

Assessment of data distribution using the Kolmogorov-Smirnov test demonstrated non-normality; therefore, non-parametric statistical methods were applied throughout the analysis.

## RESULTS

During the study period, 225 pregnant women were initially identified. Of these, 46 (20.4%) were excluded due to missing data, resulting in a final analytical sample of 179 fetuses (79.6%). The final cohort comprised 124 fetuses from women with SLE (69.3%) and 55 fetuses from women without autoimmune disease (30.7%).

All included fetuses (n=179, 100%) were evaluated using three fetal growth reference standards—Hadlock, Intergrowth-21st, and WHO—to determine EFW percentiles. Variation was observed among the reference standards in the classification of fetal growth and in the identification of FGR.

**Figure 2:**
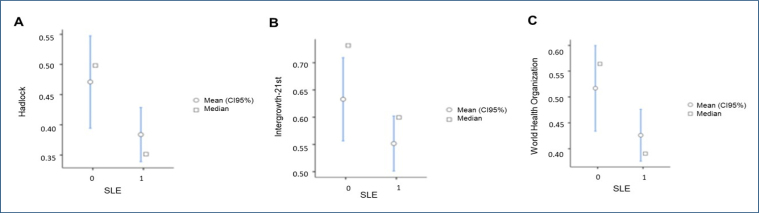
Comparison of mean and median estimated fetal weight percentiles between fetuses of women with systemic lupus erythematosus and fetuses of women without autoimmune disease, according to three fetal growth reference standards: (A) Hadlock, (B) INTERGROWTH-21st, and (C) World Health Organization.

When comparing fetal weight percentiles between groups, fetuses from women without autoimmune disease consistently exhibited higher percentile values than those from women with SLE across all three growth standards. Using the Hadlock reference, the mean fetal weight percentile was 47.1% in the control group and 38.4% in the SLE group. According to the Intergrowth-21st standard, mean percentiles were 63.3% in the control group and 55.2% in the SLE group. Using the WHO reference, mean percentiles were 51.7 and 42.6% in the control and SLE groups, respectively (Figure 2).

**Figure 3:**
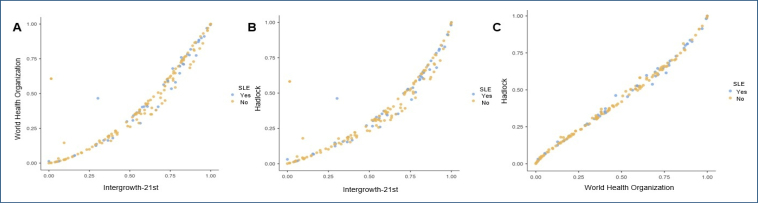
Scatter plots illustrating the relationship between estimated fetal weight percentiles derived from different fetal growth reference standards in the study population: (A) World Health Organization versus INTERGROWTH-21st, (B) Hadlock versus INTERGROWTH-21st, and (C) Hadlock versus World Health Organization. Each point represents an individual fetus.

No statistically significant differences were observed among the three fetal growth standards. However, the Intergrowth-21st reference consistently yielded higher fetal weight percentiles in both groups when compared with the Hadlock and WHO standards (Figure 3).

## DISCUSSION

The comparison among the three reference standards for fetal weight assessment (Hadlock, Intergrowth-21st, and WHO) in fetuses of pregnant women with and without SLE revealed subtle yet consistent differences in percentile classification between the studied populations. Although no statistically significant differences were observed among the three methods, the observed trends provide relevant insights for clinical interpretation and for selecting the most appropriate reference standard in specific clinical contexts.

Overall, fetuses of women without autoimmune disease exhibited higher mean percentile values across all evaluated growth curves. This finding is consistent with the existing literature describing an increased risk of FGR in pregnancies complicated by SLE, particularly in the presence of active disease, corticosteroid use, or placental vasculopathy^9^. The most pronounced difference between groups was observed using the Hadlock reference, with mean percentiles of 47.1% among women without autoimmune disease and 38.4% among those with SLE, suggesting that this standard may classify fetuses at relatively lower percentiles in pregnancies complicated by SLE.

In contrast, the Intergrowth-21st model yielded higher mean percentiles in both the SLE and non-SLE groups (55.2 and 63.3%, respectively). This pattern suggests that the Intergrowth-21st reference yields higher percentile values when compared with the other evaluated standards. It may reduce the sensitivity of Intergrowth-21st for detecting FGR, particularly in high-risk pregnancies such as those affected by SLE.

The WHO growth curve demonstrated intermediate percentile values in both the SLE (42.6%) and non-SLE (51.7%) groups, with intermediate percentile values relative to the other standards. This pattern may reflect greater suitability of the WHO reference for more heterogeneous populations, although it may also imply lower specificity for identifying subtle deviations in fetal growth.

Despite the lack of statistical significance, the observed differences may hold clinical relevance, especially considering the well-established effects of SLE on placental perfusion and fetal growth. The selection of an appropriate growth reference should therefore consider not only population-based averages but also accuracy in identifying adverse perinatal outcomes—one of the secondary objectives of this study. In this context, the apparent overestimation associated with Intergrowth-21st may lead to underdiagnosis of FGR, whereas the Hadlock reference, by yielding lower percentiles, may identify a greater proportion of fetuses below conventional growth thresholds.

Currently, there are no fetal growth charts specifically developed or validated for pregnancies complicated by SLE. As a result, fetal growth surveillance in women with SLE relies on standard population-based growth references. The Society for Maternal-Fetal Medicine recommends serial ultrasonographic assessment of fetal growth in this population due to the increased risk of FGR and other adverse perinatal outcomes; however, it does not endorse the use of disease-specific growth standards^2^. In clinical practice, universally accepted growth curves are therefore used to estimate fetal weight and to identify fetuses below the 10th percentile, which directly informs antenatal management and the need for intensified surveillance. Recent literature supports this approach, indicating that even in high-risk populations such as pregnancies affected by SLE, standard growth references remain the primary tools for screening and diagnosing FGR, given the lack of evidence supporting the benefit or validity of condition-specific curves^10–12^. In this context, comparing the performance and behavior of widely used fetal growth standards in pregnancies complicated by SLE is clinically relevant, as differences among these references may influence the detection of growth abnormalities and subsequent management decisions.

This study has several limitations. Its retrospective, single-center design may limit generalizability and introduce missing data, leading to the exclusion of a subset of cases. Detailed characterization of the SLE population, including disease activity, lupus nephritis status, medication use, and antiphospholipid antibody profile, was not consistently available due to limitations inherent to retrospective medical record review. Fetal biometric measurements were obtained as part of routine clinical care rather than a standardized research protocol, which may introduce measurement variability. In addition, the sample size—particularly in the control group—may have limited statistical power to detect small but clinically relevant differences among growth standards. Finally, this study was not designed to assess diagnostic accuracy for adverse perinatal outcomes, precluding definitive conclusions regarding the clinical superiority of any single fetal growth reference.

## CONCLUSION

Fetal weight percentiles differed according to the growth reference standard used in pregnancies complicated by SLE, despite the absence of statistically significant differences among the curves. Fetuses of women with SLE consistently exhibited lower percentile values than those of women without autoimmune disease across all standards. The INTERGROWTH-21st reference tended to yield higher fetal weight percentiles, suggesting potential overestimation, whereas the Hadlock standard generated lower percentiles and may be more sensitive to growth impairment in this high-risk population. These findings underscore the importance of understanding the behavior of commonly used fetal growth references in pregnancies affected by SLE and highlight the need for further prospective studies to determine which standards best identify clinically meaningful adverse perinatal outcomes in this population.

## Data Availability

The datasets generated and/or analyzed during the current study are available from the corresponding author upon reasonable request.

## References

[1] Castellanos Gutierrez AS, Figueras F, Morales-Prieto DM, Schleußner E, Espinosa G, Baños N (2022). Placental damage in pregnancies with systemic lupus erythematosus: a narrative review. Front Immunol.

[2] Silver R, Craigo S, Porter F, Osmundson SS, Kuller JA, Society for Maternal-Fetal Medicine (SMFM) (2023). Society for maternal-fetal medicine consult series #64: systemic lupus erythematosus in pregnancy. Am J Obstet Gynecol.

[3] (2021). Obstet Gynecol.

[4] Committee on Practice Bulletins—Obstetrics and the American Institute of Ultrasound in Medicine (2016). Practice bulletin no. 175: ultrasound in pregnancy. Obstet Gynecol.

[5] Aringer M, Costenbader K, Daikh D, Brinks R, Mosca M, Ramsey-Goldman R (2019). 2019 European League Against Rheumatism/American College of Rheumatology Classification Criteria for systemic lupus erythematosus. Arthritis Rheumatol.

[6] Martins JG, Biggio JR, Abuhamad A, Society for Maternal-Fetal Medicine (SMFM) (2020). Society for maternal-fetal medicine consult series #52: diagnosis and management of fetal growth restriction: (Replaces Clinical Guideline Number 3, April 2012). Am J Obstet Gynecol.

[7] Papageorghiou AT, Kennedy SH, Salomon LJ, Altman DG, Ohuma EO, Stones W (2018). The INTERGROWTH-21st fetal growth standards: toward the global integration of pregnancy and pediatric care. Am J Obstet Gynecol.

[8] Kiserud T, Benachi A, Hecher K, Perez RG, Carvalho J, Piaggio G (2018). The World Health Organization fetal growth charts: concept, findings, interpretation, and application. Am J Obstet Gynecol.

[9] Limaye MA, Buyon JP, Cuneo BF, Mehta-Lee SS (2020). A review of fetal and neonatal consequences of maternal systemic lupus erythematosus. Prenat Diagn.

[10] McDonald EG, Bissonette L, Ensworth S, Dayan N, Clarke AE, Keeling S (2018). Monitoring of systemic lupus erythematosus pregnancies: a systematic literature review. J Rheumatol.

[11] Wind M, Fierro JJ, Bloemenkamp KWM, Leeuw K, Lely AT, Limper M (2024). Pregnancy outcome predictors in systemic lupus erythematosus: a systematic review and meta-analysis. Lancet Rheumatol.

[12] Gamba A, Zen M, Depascale R, Calligaro A, Gatto M, Iaccarino L (2024). Modern management of pregnancy in systemic lupus erythematosus: from prenatal counseling to postpartum support. J Clin Med.

